# Adult Mental Health Outpatients Who Have Minor Children: Prevalence of Parents, Referrals of Their Children, and Patient Characteristics

**DOI:** 10.3389/fpsyt.2019.00163

**Published:** 2019-04-02

**Authors:** Torleif Ruud, Darryl Maybery, Andrea Reupert, Bente Weimand, Kim Foster, Anne Grant, Bjørg Eva Skogøy, Solveig O. Ose

**Affiliations:** ^1^Mental Health Services, Akershus University Hospital, Lørenskog, Norway; ^2^Institute of Clinical Medicine, University of Oslo, Oslo, Norway; ^3^School of Rural Health, Monash University, Moe, VIC, Australia; ^4^Faculty of Education, Monash University, Clayton, VIC, Australia; ^5^Faculty of Health Sciences, OsloMet—Oslo Metropolitan University, Oslo, Norway; ^6^School of Nursing, Midwifery and Paramedicine, Australian Catholic University, Sydney, NSW, Australia; ^7^NorthWestern Mental Health, Parkville, VIC, Australia; ^8^School of Nursing and Midwifery, Queens University Belfast, Belfast, United Kingdom; ^9^Nordland Hospital Trust, Bodø, Norway; ^10^Faculty of Health Sciences, UiT The Arctic University of Norway, Tromsø, Norway; ^11^Health Services Research, SINTEF, Trondheim, Norway

**Keywords:** prevalence of parents with mental illness, children of parents with mental illness, mental health outpatients, patient characteristics, needs of care, referrals

## Abstract

**Background:** A strong connection exists between parental mental illness and lifetime mental health risk for their children. Thus, it is important to determine, when parents attend for treatment for their illness, the prevalence and characteristics of parents with a mental illness and identify referral actions for their children. Previous studies indicate that 12–45% of adult mental health service patients are parents with minor children. There is a need for studies with larger sample sizes that investigate the prevalence and characteristics of parents, and factors associated with referral actions for their children.

**Method:** Data on 23,167 outpatients was drawn from a national census study across 107 Norwegian adult mental health outpatient clinics during 2 weeks in April 2013. Clinicians identified various socio-demographic characteristics of patients who were parents and referral actions for their children.

**Results:** Eight thousand thirty-five (36%) of outpatients had children under 18 years. Thirty-one percent were provided with referrals for their children and 58% were reported to have children with no need for referral. Three percent were reported to have children with unmet needs who were not referred. There were missing data on children's needs and referral actions for 8% of parents. Patients who care for minor children were more likely to be refugees, and less likely to be single, male, not own a house/apartment, and have a schizophrenia spectrum illness or substance use disorder. Children were more likely to be referred when their parent was single, with no income from paid work, low education, not owning house/apartment, poor family network, long outpatient treatment, and an individual care plan; and less likely for men with a moderate or less severe mental illness. Children were referred to child protection agencies, child and adolescent mental health services and school psychological/pedagogic services.

**Discussion:** The prevalence of outpatients with children is similar to other studies. Referrals were made for children of one third of outpatients with minor children. Needs and referrals of children was unknown for one in ten outpatients. Mental health outpatient clinics must improve procedures to identify parenting status and ascertain and act on children's needs.

## Introduction

This paper presents data from a national census of patients in adult mental health outpatient clinics in Norway. It provides prevalence of outpatients who care for children under the age of 18 years, prevalence of parents whose children are considered by clinicians to require further support and are referred to external agencies, and how parents' socio economic and clinical characteristics influence prevalence and type of referrals made by clinicians for the children. This information can be used by policy makers and managers to strengthen policy, as well as to support clinicians to better identify parents, determine their children's needs and refer the children to appropriate services.

Parental mental illness impacts on the functioning of the family unit and poses a risk to the healthy development of children. Compared to other children, those growing up with a parent with a mental illness are at risk of a range of adverse behavioural, developmental and emotional outcomes (1–3). Bell et al. (4) found that children of parents who had been hospitalized for a mental illness were much less likely to be school ready. Difficulties with schooling have also been shown in Sweden where Hjern et al. highlighted that twice as many children whose parents had been hospitalised for a mental illness had lower school results and were not able to start secondary education, compared to children without parental mental illness (5). However, it was also estimated that most of the risk was associated with three contributing social factors (needing social support, low parental education level, parental separation/divorce), or an interaction between parental illness and social problems. Foster et al. (6) identified parental functioning and parental mental illness among risk/protective factors for children according to the child.

Evidence suggests that children whose parents have a mental illness have almost double the chance of developing a mental illness themselves (5, 7). In a Swedish study of 535,000 children up to 30–35 years of age, those who grew up with substance abuse and/or mental illness in the family and who also required social welfare were identified as an extreme high-risk group. This group of children reported considerably higher levels of mortality, mental illness, substance abuse, criminality, and were recipients of social welfare benefits, while having low workforce participation (5). In Norway it is estimated that 25% of mental illnesses among adolescents, and 10% of early death, is associated with parental mental illness (8).

A recent systematic literature review found that between 12 and 45% of all patients attending adult mental services were parents (9). Four studies in the review found prevalence rates from 36 to 38% (10–13), suggesting that a substantial minority (one third) of patients using adult mental health services are parents (9). However, a limitation of these audit-style studies (12, 14) is that they commonly focus on a single regional adult mental health service with modest sample sizes. Other studies have assumed an epidemiological approach that projects findings from a representative sample to approximate the whole population (15) or utilised already collected national data sets (16). In Norway it has been estimated that 23.1% of children have a parent with a moderate or severe mental illness that may affect their daily life (8). Among children born in Sweden 1987–1989, 7.8% of minor children have a parent admitted to hospital due to psychiatric illness and/or substance abuse. Further, a survey from 2007 to 2011 found that parental mental health problems, not sufficiently severe to require hospitalizations, were relatively common, with 18.1% of parents to children aged 10–18 years reporting nervousness, anxiety or worry (5).

To the best of our knowledge only one study has examined whole-of-service data for parental mental health prevalence. In the early 2000s, Maybery et al. (16) found that in one year, 20% of adult mental health patients in the Australian state of Victoria were parents. In a follow up study, the authors examined state-wide adult mental health outpatient data over a 4 years period and found that 19–20% of almost 60,000 outpatients were parents (17). While the study provided valuable prevalence data, it failed to go further and illustrate information about the parents (such as gender, diagnosis) nor about the needs of their children. Consequently, there is a lack of whole of population knowledge about the prevalence of adult patients in mental health or addiction services who are parents caring for children. Such information would provide important data that can be used to inform service policy, intake procedure, and professional development of clinicians.

When assessing the risks to such children, it is essential to obtain information about the parent, including diagnosis, gender, socio economic context, and family networks, as well as other circumstances which may influence children's experiences and outcomes. For example, parents who have an anxiety disorder are less likely to grant their children autonomy and more likely to demonstrate lower levels of sensitivity (18), while children of parents with psychosis might be directly involved in a parent's delusions (19). After controlling for other risk factors at birth, Hammond et al. (20) found that two thirds of infants of mothers with a psychotic disorder were reported to child protection services. This was four times that of infants of mothers without a mental illness. Infants of mothers with a mood disorder were twice as likely to be referred to child protection services compared to infants of mothers without a mental illness.

The family environment, including the presence of marital discord, the presence or absence of the other parent and the availability of social support to the family may also influence the level of risk exposure to the children (21). There are also differences between the experiences and needs of mothers and fathers with a mental illness, in terms of custody arrangements and who they call on for help (22). These family circumstances have important implications for the development of risk-assessment tools and case-related decision making and accordingly need to be identified from the outset.

Other studies have considered parent and family variables when determining prevalence. A 4-year census in one Australian adult mental health service identified that a majority of female outpatients were parents (14). Nearly half were married, with around a third separated or divorced. Approximately half of parents reported a good level of social support. The most prevalent diagnoses were mood and psychotic disorders, followed by anxiety disorders. Approximately 60% of dependent children lived with parents, including 34–56% of children whose parents had a psychotic disorder. Around a third of children were identified as having child protection involvement (14). These findings are broadly consistent with those from another Australian adult cross-sector service census, with the majority of identified parents being female and over half of being single (23). In that audit, the most common parental diagnoses were psychotic and mood disorders, with ~40% of parents living with their children or another family member (23). Both audits however were limited by moderate sample sizes (average yearly sample size around 800 limited to specific catchment areas).

It is important that adult mental health services are responsive to the needs of children in these families, which at a minimum would involve identifying children, assessing their needs, and as required, referring them on to appropriate services (24–26). However, little is known about clinicians' actions in adult mental health outpatient services in relation to detection, follow up and referral of patients' children. Some small-scale qualitative studies (25, 27, 28) have shown that clinicians working in adult mental health services struggle to balance the needs of both parents and children and do not routinely refer children on to appropriate services. However, further research is required to generalise and extend these findings.

Research has clearly demonstrated the benefits of family focused practice for parents with a mental illness, their children and other family members (29, 30). Two controlled trials found that family focused approaches significantly improved mental health outcomes for parents (31, 32). A meta-analysis found that family focused preventive interventions reduced the risk of children developing the same mental illness as their parent by 40% (30). Consequently, to inform mental health service policy and practice it is important to identify the number of outpatients attending adult mental health services who are parents with minor children.

## Context of the Study

In Norway, specialized mental health services are organised in general hospital services across 19 health trusts. The division of mental health and addiction services in each health trust has inpatient and outpatient services for children and adolescents, adults, and older persons. Community mental health centres for adults include all outpatient mental health clinics, mobile teams, day units and almost half of the mental health inpatient beds in Norway (33–35). Almost all mental health services are public, but a few hospitals and CMHCs are owned by private trusts.

The Norwegian Health Personnel Act of 2010 requires health personnel to ascertain whether a patient has minor children and if so, to record this in their patient record. The law also stipulates that clinicians should talk with the patient about their children's needs and offer to give information and guidance. Within the limits of confidentiality, clinicians might invite children to be involved in conversations about their parents' illness, treatment, and the possibility of visiting the service treating their parent. In addition, the law stipulates that clinicians assess the needs of the children and refer children to relevant services such as child protection agency, CAMHS, educational-psychological services in schools and family counselling offices as required (36).

## Aims and Research Questions

The study aimed to determine the number and characteristics of adult outpatients who care for children under the age of 18, from a national outpatient census in mental health services in Norway. A further aim was to identify the prevalence of outpatients with minor children where the clinician identified a need for referral of children to an external agency.

The paper addresses the following research questions: 1. What is the prevalence of outpatients in adult mental health clinics who care for children under the age of 18 years? 2. What are the socio-demographic and clinical characteristics of these outpatients, compared to outpatients who do not care for minor children (in terms of diagnosis, gender, age, marital status, income, education level, housing, refugee status, and country of birth)? 3. For what prevalence of outpatients with minor children do clinicians identify a need for referral of children to an external agency for children, and what agencies are the children referred to? 4. What patient characteristics are associated with referral of children to external agencies?

## Materials and Methods

### Design

The design of the census was a cross-sectional study of outpatients seen by all outpatient clinics and mobile teams in adult Norwegian mental health services during 2 weeks in April 2013. The census was commissioned and financed by the Norwegian Directorate of Health. The work was undertaken by the SINTEF research foundation. The study was approved by the Regional Committee for Medical and Health Research Ethics (reg.no. 2012/848).

### Sample

The sample consisted of data on 23,167 adult outpatients seen by 107 of the 110 mental health outpatient clinics in Norway. The prevalence of outpatients included was 60% based on data from the National Patient Register indicating that the total number of outpatients during the 2 weeks was 38,904. The clinics that did not participate were small and cited a lack of time for not participating; these comprised 1% of all outpatient consultations during the 2 weeks.

### Variables

The census form included outpatients' socio-demographic data (gender, age, and marital status, main source of income, highest education, housing situation, refugee status, and country of birth), main mental diagnoses using ICD-10 (for substance use disorders secondary diagnosis is also included, as this is often listed as a secondary diagnosis for patients with both a mental and a substance use disorder), and the following questions about patients' children: (1) Does the patient care for children under 18 years? (yes/no/unknown). (2) If yes, number of children. (3) Have measures been taken to follow up any of the children? (available response being; yes; no and no need to refer; no and a need to refer; unknown). (4) What agencies were children referred to (possible to mark more than one of the listed agencies).

### Data Collection

All outpatients who had one or more consultations in 2 weeks (15–28 April 2013) were targeted. Several months prior to the census, service managers and clinicians received information about the census and the data collection procedures. Data were collected on hard copy forms. The clinicians completed one anonymous form for each outpatient. They were encouraged to invite the patients to participate in filling in the form, and 57% of the patients participated. The completed forms were returned to a data collection company, who scanned the forms and delivered data files to the project team.

### Data Analyses

Descriptive statistics, chi square testing and logit regressions were computed with STATA 15.

## Results

### Prevalence of Outpatients Who Care for Children Under the Age of 18

Of the 23,167 registered outpatients, information on gender and children was given for 22,398 (97%) of patients. A total of 8,035 (36%) of these had children under age 18, with 5,729 (71%) being female and 2,306 (29%) male.

### Characteristics of Outpatients Who Care for Children Under the Age of 18

Bivariate chi square analyses of associations between the patient characteristics in Table 1 show significant relationships between a higher prevalence of those caring for children under 18 and most of the variables under investigation: being female, age 30–49 years, higher education, income from paid work, having a spouse/partner, living with spouse/partner, owning a house/apartment, having good networks with family, and friends, fewer consultations since start of treatment, being a refugee, born outside Norway, not living in a large municipality, not having an individual care plan (required for anyone in need of long-term and coordinated services), not being on community treatment order, and having a diagnosis of moderate or less severe mental illness like anxiety disorders and moderate depression. Some of the same patterns are found in a more detailed analysis in Table 2 with a focus especially on gender and main diagnosis.

**Table 1:** Bivariate analyses of the association between patient characteristics and outpatients (*N* = 22,847) who care for children under 18.

	**No children**	**Have children**	**Missing data**	**Total**	**% with children**	**Chi-test (*x*^**2**^)**
**Gender**						*x*^2^ 360.66 (1) *p* < 0.001
Female	8,425	5,729	262	14,416	40	
Male	5,938	2,306	187	8,431	28	
**Age group**						*x*^2^ 4087.50 (6) *p* < 0.001
18–23 years	3,258	249	69	3,576	7	
24–29 years	2,919	1,029	74	4,022	26	
30–39 years	2,603	2.965	81	5,649	53	
40–49 years	1,935	2,666	93	4,694	58	
50–59 years	1,805	765	52	2,622	30	
60–69 years	850	95	20	965	10	
70 years and above	672	59	31	762	8	
**Education**						*x*^2^ 506.11 (2) *p* < 0.001
High education	2,432	2,251	60	4,743	48	
Medium education	6,289	3,597	131	10,017	36	
Low education	5,772	2,267	325	8,364	28	
**Main source of income**						*x*^2^ 784.99 (2) *p* < 0.001
Income from labour	3,164	2,922	77	6,163	48	
Health related benefit	7,762	4,179	175	12,116	35	
Other economic support	3,567	1,014	264	4,845	22	
**Marital status**						*x*^2^ 4533.88 (2) *p* < 0.001
Married/cohabitant/partner	3,712	5,203	106	9,021	58	
Separated/divorced/widower/widow	1,624	1,355	64	3,043	45	
Single/unmarried	9,061	1,466	190	10,717	14	
**Household**						*x*^2^ 4258.02 (2) *p* < 0.001
Alone with or without children	7,033	2,704	172	9,909	28	
Spouse/cohabitant	3,596	5,090	98	8,784	59	
Other household	3,705	201	79	3,985	5	
**Accommodation**						*x*^2^ 2614.39 (3) *p* < 0.001
Own house	5,156	5,306	125	10,587	51	
Rented house/apartment, private market	4,298	1,868	95	6,261	30	
Rented house/apartment, local authorities	1,466	341	27	1,834	19	
Other	3,305	361	72	3,738	10	
**Network family**						*x*^2^ 79.72 (3) *p* < 0.001
Very good	4,169	2,642	113	6,924	39	
Good	6,325	3,561	124	10,010	36	
Poor	1,998	979	43	3,020	33	
Very poor	715	251	30	996	26	
**Network friends**						*x*^2^ 178.45 (3) *p* < 0.001
Very good	3,013	2,078	65	5,156	41	
Good	6,531	3,881	152	10,564	37	
Poor	2,180	996	58	3,234	31	
Very poor	825	238	22	1,085	22	
**Number of consultations since start of treatment**						*x*^2^ 145.81 (7) *p* < 0.001
< 3	1,567	847	72	2,486	35	
3–5	1,551	1,009	37	2,597	39	
6–9	1,601	1,007	40	2,648	39	
10–19	2,345	1,381	70	3,796	37	
20–39	2,321	1,355	47	3,723	37	
40–99	2,155	1,204	43	3,402	36	
100–199	1,064	484	28	1,576	31	
200 or more	725	185	12	922	20	
**Asylum seeker**						*x*^2^ 0.67 (1) *p* = 0.4129
No	14,426	8,071	513	23,010	36	
Yes	67	44	3	114	40	
**Refugee**						*x*^2^ 36.22 (1) *p* < 0.001
No	14,093	7,768	496	22,357	36	
Yes	400	347	20	767	46	
**Born outside Norway**						*x*^2^ 94.51 (1) *p* < 0.001
No	12,973	6,903	466	20,342	35	
Yes	1,520	1,212	50	2,782	44	
**Having an individual care plan**						*x*^2^ 411.4 (1) *p* < 0.001
No	12,329	7,608	471	20,408	38	
Yes	2,164	507	45	2,716	19	
**Community treatment order**						*x*^2^ 193.3 (1) *p* < 0.001
No	13,866	8,019	500	22,385	37	
Yes	627	96	16	739	13	
**Living in a large municipality**						*x*^2^ 244.16(1) *p* < 0.001
No	10,680	6,709	413	17,802	39	
Yes	3,813	1,406	103	5,322	27	
**Diagnoses**						*x*^2^ 1013.38(7) *p* < 0.001
Personality disorders	1,118	640	33	1,791	36	
Substance use disorders (as first or second diagnosis)	1,058	241	27	1,326	19	
Schizophrenia etc.	1,902	295	52	2,249	13	
Affective disorders	3,982	2,690	94	6,766	40	
Anxiety disorders	3,128	2,455	90	5,673	44	
Behavioural syndromes	552	221	14	787	29	
Behavioural and emotional disorders	557	418	16	991	43	
Other mental illness	1,320	619	27	1,966	32	

**Table 2:** Gender, diagnosis and prevalence of outpatients (*N* = 8,035) with care for children under 18 among sociodemographic subgroups.

	**Number of patients with children**	**Patients with children (%)**	**Type of household (%) with or without children**			**Poor family network (%)**	**Poor friend network (%)**
			**Alone**	**Spouse**	**Other**		
**Gender**							
Female	5,729	40	41	43	15	17	16
Male	2,306	28	47	31	21	18	24
**Diagnoses**							
Personality disorders	635	37	49	38	13	30	29
Substance use disorders (as first or second diagnosis)	240	19	61	19	20	27	30
Schizophrenia etc.	293	13	67	14	18	19	29
Affective disorders	2,669	40	42	44	14	16	16
Anxiety disorders	2,429	44	36	49	15	15	15
Behavioural syndromes	219	29	33	37	30	12	13
Behavioural and emotional disorders	412	43	43	36	21	15	13
Other mental illness	613	32	36	37	27	15	18

In a logistic regression analysis of associations between the odds ratio for caring for children under 18 and the same patient characteristics (Table 3), most of the patient characteristics showed a significant association with the same direction as in the bivariate analyses, but with various odds ratios. The significant odds ratios were highest for age groups 30–49 and refugees; and lowest for being single, male, not having own house/apartment, and having a schizophrenia spectrum illness, or substance use disorder.

**Table 3:** Logistic regression of the association between patient characteristics and whether the outpatients (*N* = 22,847) care for children under 18.

	**Odds ratio**	**Std. Err**.	**z**	***P* > *z***	**95% Confidence interval**	
**Gender**						
Male	0.676	0.028	−9.37	0.00	0.623	0.734
**Age group**						
18–23 years	1.000	(base)				
24–29 years	3.029	0.274	12.25	0.00	2.537	3.617
30–39 years	8.114	0.724	23.46	0.00	6.812	9.665
40–49 years	7.821	0.730	22.02	0.00	6.512	9.392
50–59 years	1.666	0.168	5.07	0.00	1.367	2.029
60–69 years	0.349	0.051	−7.21	0.00	0.262	0.464
70 years and above	0.213	0.037	−8.80	0.00	0.151	0.300
**Education**						
High education	1.000	(base)				
Medium education	1.033	0.050	0.67	0.51	0.939	1.136
Low education	1.167	0.065	2.75	0.01	1.046	1.303
**Income**						
Income from labour	1.000	(base)				
Health related benefits	0.857	0.039	−3.36	0.00	0.784	0.938
Other economic support	1.062	0.071	0.89	0.37	0.930	1.211
**Marital status**						
Married/cohabitant/partner	1.000	(base)				
Separated/divorced/widow/widower	0.842	0.117	−1.23	0.22	0.641	1.106
Single/unmarried	0.168	0.023	−12.99	0.00	0.128	0.220
**Type of household**						
Alone with or without children	1.000	(base)				
Spouse/cohabitant	0.832	0.113	−1.35	0.18	0.637	1.086
Other type of household	0.298	0.031	−11.67	0.00	0.243	0.365
**Accommodation**						
Own house	1.000	(base)				
Rented house/apartment, private marked	0.647	0.031	−9.10	0.00	0.589	0.710
Rented house/apartment, local authorities	0.564	0.049	−6.60	0.00	0.476	0.669
Other accommodation	0.638	0.058	−4.92	0.00	0.534	0.763
**Poor network family**	0.906	0.048	−1.88	0.06	0.817	1.004
**Poor network friends**	0.820	0.043	−3.79	0.00	0.740	0.909
**Consultations 20 or more since start**	0.870	0.035	−3.49	0.00	0.805	0.941
**Refugee**	1.399	0.163	2.87	0.00	1.112	1.759
**Born outside Norway**	1.156	0.074	2.27	0.02	1.020	1.311
**Has and Individual care plan**	0.731	0.051	−4.53	0.00	0.638	0.837
**Community treatment order (CTO)**	0.838	0.120	−1.24	0.22	0.633	1.109
**Living in large municipality**	0.681	0.032	−8.20	0.00	0.622	0.747
**Main diagnoses**						
Personality disorders	1.000	(base)				
Substance use disorders (as first or second diagnosis)	0.627	0.068	−4.31	0.00	0.507	0.775
Schizophrenia spectrum disorders	0.406	0.041	−8.90	0.00	0.333	0.495
Affective disorders	1.047	0.074	0.65	0.52	0.911	1.204
Anxiety disorders	1.038	0.075	0.52	0.60	0.901	1.197
Behavioural syndromes	0.718	0.088	−2.71	0.01	0.565	0.912
Behavioural and emotional disorders	1.528	0.164	3.95	0.00	1.238	1.886
Other mental illness	1.083	0.101	0.85	0.39	0.902	1.301
Constant	0.712	0.129	−1.88	0.06	0.500	1.015

### Prevalence of Outpatients With Minor Children Being Referred

The data on the number of children identified by clinicians to require referral to an external agency is shown in Table 4.

**Table 4:** Reported needs and whether measures have been taken to follow up of the children of outpatients (*N* = 8,035) with care for children under 18.

	**Number of patients**	**% of patents**
Yes child/ren have needs	2,488	31
No, but have needs	247	3
No, but does not have any needs	4,670	58
Do not know	257	3
No answer	373	5
Number of outpatients with responsibility for children under age 18	8,035	100

The clinicians answered the question on whether measures have been taken for referral for the children for 7,405 (92%) outpatients. Of these, 2,488 (31%) were reported to require referral to an agency. Of the 4,917 (61%) outpatients with children reported as not being referred, 247 (3%) were still reported to need a referral. This indicates that for 34% of the parents a referral of their children was identified as required, that some of these were not referred, and that children of 58% of the parents were reported to not require a referral. The need for referral is unknown for children of 630 (8%) of outpatients with minor children, including those who responded with “Do not know” and those with missing answers. Patients were involved in filling in the form in 61% of cases where the patient was a parent (*n* = 8,035). Sixty-three percent of female patients contributed to filling in the form compared to 59% of the male patients. If the parent was involved in filling in the form, 2% of the answers regarding measures taken for referrals were “Don't know” compared to 6% if the parent was not involved.

Information on the referral agencies is presented in Table 5. Almost half the outpatients with referred children (45%) had children who were referred to child protection agencies, closely followed by child and adolescent mental health services (39%) and educational-psychological services in the school system (35%). The most common combinations of services involved in follow up of the children are adult mental health outpatient clinics together with family counselling agencies (*n* = 388) or child protection agencies (*n* = 304).

**Table 5:** Prevalence of outpatients (*N* = 2,488) where children are reported to be followed up by various services or agencies for children^*^.

	**Number of patients**	**% of patients**
Child protection agency	1,128	45
Family counselling office	165	7
Educational-psychological service/ school	861	35
Child and adolescent mental health service	975	39
Adult mental health outpatient clinic	83	3
Other	544	22
No answer	46	2

* *More than one agency may have been marked for each outpatient*.

### Characteristics for Outpatients With Minor Children Being Referred

Bivariate chi square analyses of associations between the patient characteristics and whether measures were taken to refer children are shown in Table 6. There are highly significant associations for gender, age group, level of education, main source of income, marital status, type of household, accommodation, networks of family, network of friends, number of consultations since start of treatment, having an individual care plan, being under community treatment order, and main diagnosis. There are no significant associations for the size of the municipalities or for being part of a minority group (asylum seeker, refugee, born outside Norway). The pattern of significant differences shows that many indicators on lower socio-demographic status as well as severe mental illness and substance use disorder are associated with referrals of children.

**Table 6:** Bivariate analyses of characteristics of outpatients with children (*N* = 8,035)^*^ and whether measures have been taken to follow up their children.

	**No follow up**	**Follow up**	**Missing**	**Total**	**% Follow up**	**Chi-test (*X*^**2**^)**
**Gender**						*x*^2^ 127.13 (1) *p* < 0.001
Female	3,479	1,977	273	5,729	35	
Male	1,695	511	100	2,306	22	
**Age group**						*x*^2^ 59.88 (6) *p* < 0.001
18–23 years	169	67	11	247	27	
24–29 years	673	305	43	1,021	30	
30–39 years	1,839	959	135	2,933	33	
40–49 years	1,661	847	138	2,646	32	
50–59 years	530	213	21	764	28	
60–69 years	79	11	4	94	12	
70 years and above	55	3	1	59	5	
**Education**						*x*^2^ 148.97 (2) *p* < 0.001
High education	1,631	508	89	2,228	23	
Medium education	2,303	1,111	150	3,564	31	
Low education	1,240	869	134	2,243	39	
**Main source of income**						*x*^2^ 154.09 (2) *p* < 0.001
Income from labour	2,107	658	126	2,891	23	
Health related benefit	2,460	1,494	187	4,141	36	
Other economic support	607	336	60	1,003	33	
**Marital status**						*x*^2^ 197.22 (2) *p* < 0.001
Married/cohabitant/partners	3,587	1,319	250	5,156	26	
Separated/divorced/widower/widow	723	564	51	1,338	42	
Single/unmarried	807	580	66	1,453	40	
**Household**						*x*^2^ 180.19 (2) *p* < 0.001
Alone with or without children	1,472	1,094	110	2,676	41	
Spouse/cohabitant	3,498	1,304	243	5,045	26	
Other household	133	56	8	197	28	
**Accommodation**						*x*^2^ 186.72 (3) *p* < 0.001
Own house	3,621	1,386	247	5,254	26	
Rented house/apartment, private marked	1,044	732	73	1,849	40	
Rented house/apartment, local authorities	149	182	7	338	54	
Other	216	124	17	357	35	
**Network family**						*x*^2^ 100.54 (3) *p* < 0.001
Very good	1,817	685	115	2,617	26	
Good	2,266	1,097	164	3,527	31	
Poor	527	404	36	967	42	
Very poor	129	114	5	248	46	
**Network friends**						*x*^2^ 41.07 (3) *p* < 0.001
Very good	1,430	543	82	2,055	26	
Good	2,430	1,244	173	3,847	32	
Poor	581	359	40	9,80	37	
Very poor	144	83	10	237	35	
**Number of consultations since start of treatment**						*x*^2^ 109.68 (7) *p* < 0.001
Less than 3	578	215	48	841	26	
3–5	684	259	51	994	26	
6–9	670	274	56	1,000	27	
10–19	912	374	78	1,364	27	
20–39	856	435	50	1,341	32	
40–99	720	437	37	1,194	37	
100–199	271	199	11	481	41	
200 or more	80	100	4	184	54	
**Asylum seeker**						*x*^2^ 0.59 (1) *p* = 0.4413
No	5,148	2,472	372	7,992	31	
Yes	26	16	1	43	37	
**Refugee**						*x*^2^ 2.35 (1) *p* = 0.1252
No	4,942	2,395	357	7,694	31	
Yes	232	93	16	341	27	
**Born outside Norway**						*x*^2^ 272 (1) *p* = 0.0993
No	4,427	2,093	316	6,836	31	
Yes	747	395	57	1199	33	
**Having an individual care plan**						*x*^2^ 154.62 (1) *p* < 0.001
No	4,971	2,198	362	7,531	29	
Yes	203	290	11	504	58	
**Community treatment order**						*x*^2^ 13.11 (1) *p* < 0.001
No	5,128	2,441	370	7,939	31	
Yes	46	47	3	96	49	
**Living in a large municipality**						*x*^2^ 0.49(1) *p* = 0.4856
No	4,280	2,042	322	6,644	31	
Yes	894	446	51	1,391	32	
**Diagnoses**						*x*^2^ 107.14(7) *p* < 0.001
Personality disorders	353	261	21	635	41	
Substance use disorders (as first or second diagnosis)	110	119	11	240	50	
Schizophrenia etc.	166	115	12	293	39	
Affective disorders	1,769	785	115	2,669	29	
Anxiety disorders	1,648	661	120	2,429	27	
Behavioural syndromes	152	55	12	219	25	
Behavioural and emotional disorders	235	161	16	412	39	
Other mental illness	385	191	37	613	31	

* *Those who receive follow up (n = 2,488) compared to those who do not (n = 5,174) or we do not know (n = 373)*.

Results of a logistic regression of the association between patient characteristics and odds ratios for referral of children is shown in Table 7 for the 6,634 outpatients with non-missing data for the independent variables in the logistic regression and who had children who did or did not receive a referral. The significant odds ratios were highest for age groups 30–59, lower education, being single, not having income from paid work, not owning house/apartment, poor family network, having had many outpatient consultations, and having an individual care plan. The significant odds ratios were lowest for being male and for having moderate or less severe mental illness.

**Table 7:** Logit regression of the association between patient characteristics and whether minor children of outpatients (*N* = 6634)^*^ are being referred.

	**Odds ratio**	**Std. Err**.	**z**	***P* > *z***	**95% Confidence interval**	
**Gender (male)**	0.466	0.032	−11.060	0.00	0.407	0.533
**Age group**						
18–23 years	1.000	(base)				
24–29 years	1.316	0.237	1.53	0.13	0.925	1.873
30–39 years	1.986	0.343	3.97	0.00	1.416	2.785
40–49 years	1.952	0.345	3.78	0.00	1.380	2.761
50–59 years	1.702	0.332	2.73	0.01	1.161	2.495
60–69 years	0.449	0.175	−2.05	0.04	0.209	0.966
70 years and above	0.123	0.092	−2.79	0.01	0.028	0.535
**High education**	1.000	(base)				
Medium education	1.419	0.102	4.87	0.00	1.233	1.634
Low education	1.896	0.156	7.77	0.00	1.613	2.228
**Income from labour**	1.000	(base)				
Health related benefits	1.376	0.091	4.83	0.00	1.209	1.566
Other economic support	1.361	0.136	3.07	0.00	1.118	1.657
**Married/cohabitant/partner**	1.000	(base)				
Separated/divorced/widow/widower	2.534	0.512	4.60	0.00	1.705	3.766
Single/unmarried	2.056	0.423	3.51	0.00	1.374	3.076
**Alone with or without children**	1.000	(base)				
Spouse/cohabitant	1.427	0.284	1.79	0.07	0.966	2.107
Other type of household	0.562	0.125	−2.58	0.01	0.362	0.870
**Own house**	1.000	(base)				
Rented house/apartment, private marked	1.337	0.097	4.02	0.00	1.161	1.541
Rented house/apartment, local authorities	1.701	0.236	3.83	0.00	1.296	2.232
Other	1.439	0.233	2.25	0.03	1.047	1.978
**Poor network family**	1.447	0.112	4.79	0.00	1.244	1.683
**Poor network friends**	1.032	0.083	0.40	0.69	0.882	1.209
**Consultations from start: 20 or more**	1.281	0.075	4.23	0.00	1.142	1.436
**Having an individual care plan**	2.232	0.247	7.27	0.00	1.797	2.771
**Community treatment order**	1.121	0.297	0.43	0.67	0.667	1.884
**Main diagnosis**						
Personality disorders	1.000	(base)				
Substance use disorders (as first or second diagnosis)	1.306	0.239	1.46	0.14	0.913	1.870
Schizophrenia spectrum disorder	0.857	0.147	−0.90	0.37	0.612	1.198
Affective disorders	0.774	0.080	−2.48	0.01	0.633	0.947
Anxiety disorders	0.684	0.072	−3.64	0.00	0.557	0.839
Behavioural syndromes	0.606	0.116	−2.61	0.01	0.416	0.883
Behavioural and emotional disorders	1.174	0.172	1.09	0.27	0.880	1.565
Other mental illness	1.124	0.155	0.85	0.40	0.858	1.472
Constant	0.099	0.028	−8.05	0.00	0.056	0.174

* *One thousand twenty-eight patients had missing values for one or more of the variables, see Table 6*.

## Discussion

In the current study one third of adult outpatients from 107 Norwegian mental health outpatient clinics cared for children under 18 years of age. One third of those parents had children who required a referral, six out of 10 had children not requiring a referral, and for one in 10 parents, the needs of their children was unknown or not reported. Children of three of 10 outpatient parents were reported to have been referred to relevant services. Patient characteristics associated with referral actions for their children were low education, being single, not owning a house/apartment, having a poor family network, having an individual care plan, being female, and having moderate or a less severe mental illness.

### Prevalence of Outpatients Who Care for Children Under 18

Thirty-six percent of the outpatients in this study cared for children under 18 years of age. This falls within the range of 36–38% found in four previous studies identified in a systematic review of adult mental health services (9). Importantly, this study is the first of its kind internationally to illustrate “whole of country” population data and represents a significant step forward in parent prevalence statistics. The results provide a strong rationale for intake systems to identify parenting status, incorporate parenting roles, and responsibilities in treatment plans, and assess and address the needs of patients' children. Overall, these data confirm that a significant minority of patients in adult mental health services are parents with children under the age of 18.

### Characteristics of Outpatients With Care for Minor Children

The results also provide greater certainty about the sociodemographic characteristics of parents and children in mental health services. Sociodemographic patient characteristics associated with higher odds ratios for caring for children under 18 were being age groups 30–49 and refugee, and characteristics associated with lower odds ratios were being male, single, not having own house/apartment, poor network of friends and not living in a large municipality. Forty percent of females and 28% of males cared for minor children. In terms of gender, others have shown somewhat similar results, with between 34 and 59% of all female patients recorded as mothers and 25–39% of all males as fathers (10, 13). A 4-year census in one Australian adult mental health service found nearly half of parents (41.2–45.0%) were married, with around a third (30.8–36.2%) separated or divorced. Approximately half of parents (43.9–52.9%) reported a good level of social support. Having a good level of support and network of friends is important for those with a mental illness and especially for the well-being of parents and children (37). A Norwegian study (38) found that the prevalence of refugees was higher among the outpatients in a mental health clinic than in the population in the catchment area, but still concluded that the outpatient clinic was probably underused by refugees based on much higher self-reported mental health problems among refugees that others in an epidemiological study in the area. The higher odds ratio for refugees being parents in the current study may be due to a higher prevalence of refugees being parents to minors, as shown in Table 1.

Clinical patient characteristics associated with lower odds ratio for caring for children under 18 were longer outpatient care, having an individual care plan, and having a severe mental illness or a substance use disorder. These data would indicate that those with a severe illness by and large are not presenting to adult mental health services with children or are not disclosing that they have children. It might also be that those with severe mental illness are more often hospitalized and/or cared for by the community care teams than by mental health outpatient clinics, and that more parents with severe mental illness might have lost custody of their children and have little contact with the children. The fear of the involvement of child protection agency might dissuade patients who have a severe illness from disclosing their parenting status (39) and the data highlighting referral patterns in this study in some ways substantiates these fears.

Forty-four percent of parents were reported to have an anxiety disorder, 40% an affective disorder, 43% a behavioural and emotional disorder, and 37% a personality disorder. In Australia, Fernbacher et al. (23) found that 42% of parents in an outpatient service had schizophrenia, 23% depression, and 13% bipolar disorder. In the 4-year census in one Australian adult psychiatric service (14) the most prevalent diagnoses were mood (35–42%) and psychotic (22–35%) disorders, followed by anxiety (11–20%) disorders. Overall, it appears that many parents in adult mental health services have an affective disorder. This is important information that can be used to inform treatment as well as continued professional development for clinicians regarding family focused practice.

### Prevalence of Outpatients With Minor Children Being Referred

For 92% of the outpatients with minor children the clinicians had identified if children required a referral to an external agency, and the need for referral was not ascertained for 8% of outpatients with minor children. Children of 61% of outpatient parents were not considered by clinicians to require a referral, even though 3% of these were still provided with a referral to an external agency. In Norway health personnel are required to ascertain whether a patient has minor children, talk with the patient about their children's needs and offer to give information and guidance. But there are no standard procedures or rules for referral of the patients' children. There may be capacity problem in the child and welfare services, as well as variations in resources and availability of such services. However, as shown in Table 6, we did not find significant differences between large and small municipalities in the probability of measures taken to follow up children.

Clinicians' actions did not necessarily align with their stated beliefs, an incongruence that has also been highlighted elsewhere (27). Altogether 34% of outpatients with minor children had children who had been referred. For 58% of outpatients, their children were identified as not requiring any further referral or support. In one Australian audit around a third (28–39%) of all children had been identified as requiring the involvement of child protection services (14), slightly less than what was found in the current study (45%). Both figures however indicate sizable referrals to child protection agencies which highlights the need for collaborative service delivery models but also the important role that child protection plays in the lives of these families.

Given the range of risk and protective factors for these children, not all children whose parent has a mental illness will be adversely affected (40), nor will all children in the same family be affected in the same way. Nonetheless, given the high risks for children associated with parental mental illness it is incumbent on clinicians to provide appropriate assessment and monitor their needs over time. It is not clear from the present study whether clinicians undertake such an assessment or whether, as some studies have shown (27), they did not always acknowledge children's needs and accordingly did not follow through on appropriate referrals. Future open-ended survey responses and/or qualitative research may be needed to identify barriers and facilitators to these clinical decisions. In a Norwegian cross-sectional study including a questionnaire to both patients with children and to clinicians in mental health services, 95% of the clinicians answered that they had talked with the patient about the situation with their children, while 71% of patients said that they had such a conversation with a clinician (41).

### Characteristics for Outpatients With Minor Children Being Referred

Sociodemographic patient characteristics associated with higher odds ratio for children being referred were being female, single, low education, not owning a house/apartment, not receiving income from paid work, and a poor family network. Maybery et al. (16) argue that risks for children escalates when the parent is single, without a support network, experiences housing insecurity and lives in poverty. So, it is perhaps not surprising that these children were more likely to be referred than other children. However, the specific reasons for why these children were referred still remains unclear.

Having an individual care plan was associated with higher odds ratio for children being referred, while moderate to less severe mental illness was associated with lower odds ratio for children being referred. Though the odds ratio of children being referred were parents having a moderate to less severe psychiatric illness, the established individual care plan suggests that these are families with multiple problems, with a need for coordinated support. The poorer family network might also be an important explanation for the higher need for other types of support.

For 52% of the patients with minor children, children had been referred to child protection agencies (45%) and family counselling offices (7%). This is not surprising as the sociodemographic characteristics of the patients with children being referred might indicate a need for economic and practical help and social support as well as help to cope with the parental illness. Many children (39%) had been referred to the child- and adolescent services, indicating that the children needed treatment for their own mental and/or behavioral problems.

For one third (35%) of the families, children had been referred to educational-psychological services/school, which confirms the need for support found in a Swedish study. Hjern et al. (5) argue that both parents' illness and substance abuse negatively impact school performance in their children, with social factors also having an important impact, and preventive general interventions in schools should be establish for such children. This would apply to children with parental illness, as well as children with separated/divorced parents or parents with low educational levels.

Some explanatory variables in the logistic regression in Table 7 are correlated, but not to the extent that this is problematic. The highest correlation is between the variable accommodation and marital status. If accommodation is removed from the regression, the size of the coefficient of marital status is the same as when accommodation is included.

In this study, if the patient did not participate in filling in the form, the question on whether children are referred was more often (6%) answered with unknown compared to if the patient participated in filling in the form (2%). This supports the importance of clinicians involving the patient in assessing the needs of their children.

### Strengths and Limitations

A major strength of the study is the large census-level sample from almost all the psychiatric outpatient clinics in one country. As the data was registered on anonymous forms and did not require written consent from the patients, there is no obvious reason to expect that the material is skewed due to subgroups being less reluctant to participate. Some clinicians did however indicate that completing the form was time-consuming and that they did not have time to include all their patients. A limitation is that the clinicians might have only included the less complicated cases to save time. It is also possible that outpatients who missed their appointed consultations were less likely to be included. An important limitation of the study compared with epidemiological studies is that only those who receive treatment were included. The prevalence numbers presented are valid for outpatients in specialist mental health treatment and not for the population suffering from mental illness in general. Finally, the results may not be representative of patients in other types of mental health services and in other countries.

The amount of missing data is a limitation, as is the small amount of information collected regarding patients' children. Information was only collected on whether children were considered to have needs and whether these needs warranted a referral to external agencies. It was not possibility to examine the quality and thoroughness of these assessments. Information was not collected on what children's needs were, nor whether referral actions were appropriate, implemented and ultimately successful at addressing children's needs. Future research could explore these factors.

## Conclusions and Implications

### Conclusions

The prevalence of outpatients in adult psychiatric clinics who care for children under the age of 18 years is 36% in this study of a sample from a national census in Norwegian adult mental health clinics. This provides considerable certainty regarding parent prevalence as the statistics emanate from a whole of country data set. The findings also add important information about the characteristics of higher risk families. Significant odds ratios among psychiatric outpatients being parents with care for minor children were highest for age groups 30–49 and refugees; and lowest for being single, male, not having own house/apartment, and having a schizophrenia spectrum disorder. Of the 92% of the patients with minor children where clinicians had answered whether measures have been taken for referral for the children, 31% were reported to have children having been referred, 58% did not have children requiring a referral, and 3% had children who had not been referred in spite of being identified as requiring a referral. The need for referral was unknown for children of 8% of the outpatients with minor children. The agencies most referred to were child protection agencies (45%), CAMHS (39%) and educational-psychological services in the school system (35%). The significant odds ratios for having children who required referral to an agency were highest for parents aged 30–59 who were single with low education, not having income from paid work, not owning a house/apartment, poor family network, having had many outpatient consultations, and having an individual care plan. The significant odds ratios were lowest for males and for having moderate or less severe psychiatric illness.

### Service and Practice Implications

There are several practice and systems implications for the present results. Given the sizable minority of patients who are parents, adult mental health services require appropriate infrastructure systems and procedures to identify parenting status of patients, including those who may be pregnant. Treatment plans should address needs of parents and their children and include referral of parents and their children to early intervention services as appropriate. Referrals to child protection agencies should be carefully considered, once accurate and sensitive assessments of the family's strengths as well as vulnerabilities are made, including the family's personal and professional networks. Appropriate training could be offered to clinicians in adult mental health outpatient services, with particular attention on how mental illness impacts parenting and the range of services that families may be referred to.

### Implications for Research

Future studies need to investigate more broadly the family circumstances for children whose parents have mental illness, the specific actions of clinicians and the outcome of referrals for children. Why clinicians provide referrals for the children of some of their patients, but not others, could be further investigated. The pathways of care, as parents and children navigate various mental health and other systems (including but not limited to adult mental health, child protection services, schools), could be documented to determine what happens for families, areas of duplication/gaps, and outcomes for different family members, over time.

## Author Contributions

TR, DM, and SO contributed conception and design of the study with input from AR, KF, BW, BS, and AG. SO collected the data, organized the database and performed the statistical analyses. DM and TR wrote the first draft of the manuscript. AR, KF, BW, BS, AG, and SO wrote sections of the manuscript. All authors contributed to manuscript revision, read and approved the submitted version.

### Conflict of Interest Statement

The authors declare that the research was conducted in the absence of any commercial or financial relationships that could be construed as a potential conflict of interest.
